# Docosahexaenoic acid ameliorates palmitate-induced lipid accumulation and inflammation through repressing NLRC4 inflammasome activation in HepG2 cells

**DOI:** 10.1186/1743-7075-9-34

**Published:** 2012-04-19

**Authors:** Xiaoqin Luo, Yan Yang, Tianran Shen, Xilan Tang, Yunjun Xiao, Tangbin Zou, Min Xia, Wenhua Ling

**Affiliations:** 1 Guangdong Provincial Key Laboratory of Food, Nutrition and Health, School of Public Health, Sun Yat-sen University, Guangzhou, China; 2 Department of Nutrition, School of Public Health, Sun Yat-sen University, 74 Zhongshan Road II, Guangzhou, 510080, Peoples Republic of China

**Keywords:** Docosahexaenoic acid, Inflammation, Lipid accumulation, Nonalcoholic steatohepatitis, NLRC4 inflammasome, Palmitate

## Abstract

**Background:**

N-3 polyunsaturated fatty acids, such as docosahexaenoic acid (DHA; 22:6n-3), has clinical significance in the prevention and reversal of nonalcoholic steatohepatitis (NASH). However, the precious mechanism underlying remains unclear. The inflammasome, a multiprotein complex formed by NOD-like receptor (NLR) family members, has been recently shown to be activated in NASH and promote the cleavage of the pro-inflammatory cytokines to their maturation forms.

**Methods:**

HepG2 cells were exposed to different dose of PA for 24 h with or without the preincubation of 50 μM DHA for another 24 h and then lipid deposition was assessed with Oil red O staining and intracellular triglyceride (TG) determination. Secretory levels of inflammatory cytokines and Caspase-1 activity were determined by ELISA assays. Gene expression and protein levels were determined by quantitative RCR and western blotting, respectively.

**Results:**

Palmitate (PA) dose-dependently increased lipid accumulation, TG content and induced the secretion of interleukin-1β (IL-1β), IL-18, TNF-α and MCP-1 from HepG2 cells. Preincubation with DHA significantly alleviated PA-induced lipid accumulation and inflammatory agents. DHA was also found to attenuate PA-induced NOD-like receptor protein 4 (NLRC4) mRNA expression. Furthermore, PA induced caspase-1 activation in a dose-dependent manner, resulting in exacerbating of procaspase-1 and pro-IL-1β processing. Knockdown of NLRC4 partially abrogated PA-induced caspase-1 activation and IL-1β maturation and completely abolished these events in the presence of DHA.

**Conclusions:**

Our findings indicate DHA attenuates PA-induced lipid accumulation and inflammation through suppressing NLRC4 inflammasome activation, caspase-1 activation and IL-1β cleavage.

## Background

Non-alcoholic fatty liver disease (NAFLD) is a worldwide common liver disease that has an increasing prevalence not only in adults with metabolic syndrome but also in obese children and adolescents [1-3]. Non-alcoholic steatohepatitis (NASH) is one of pathologic stages of NAFLD and the main histopathological changes are steatosis, inflammation and hepatocyte injury with or without fibrosis [4]. Although the precise mechanisms need to be elucidated, the two-hit hypothesis of NASH has been widely accepted [5]. The 1^st^ hit involves fat accumulation in the liver as a result of excessive delivery of free fatty acids (FFAs) from the adipose tissue, and imbalance of lipid synthesis and export in hepatocyte. The consequent injures such as oxidative stress and inflammation are regarded as the 2^nd^ hit culminating in fibrosis of liver.

Despite abundant evidences accumulated for a role of inflammatory mediators in NASH, less is known about how the inflammatory mediators are generated. Recent studies implicated that the inflammasomes have emerged as pivotal sensors of infection and stress in intracellular compartments and appear to regulate the production of the proinflammatory cytokines IL-1β and IL-18 [6,7]. It has been demonstrated that one of the NOD-like receptor (NLR) family members, NOD-like receptor protein 3 (NLRP3) inflammasome, was involved in caspase-1 activation and caspase-1-mediated IL-1β and IL-18 release [8]. However, the role of another inflammasome NOD-like receptor protein 4 (NLRC4) on the generation of inflammatory mediators in NASH has not been elucidated.

Many studies have suggested that FFAs composition and abnormal fatty acid metabolism have been implicated in the pathogenesis of NASH [9-12]. Diets with a high intake of fat, especially saturated fatty acids, may promote the development of NASH [13,14]. Conversely, polyunsaturated fatty acids (PUFAs), such as docosahexaenoic acid (DHA), have been found to decrease liver fat content in children with NAFLD and possess anti-inflammatory effects [15,16]. It has been reported that PA induced the activation of the NLRP3 inflammasome which then senses obesity-associated danger signals and contributes to obesity-induced inflammation [17,18]. However, the effects of DHA on NLRC4 inflammasome in PA-induced inflammatory responses remain unknown. In the present study, our findings demonstrated that NLRC4 inflammasome is activated in PA-induced lipid accumulation and inflammation; and that DHA alleviates these events by suppressing the activity of caspase-1 and the cleavage of procaspase-1 and pro-IL-1β through inhibiting NLRC4 inflammasome activation.

## Methods

### Materials

DHA (Sigma, St. Louis, MO) was dissolved in ethanol (stock solution 100 mM). Stock solutions were kept at −20°C before the experiments. PA (Sigma, St. Louis, MO) was conjugated with FFA-free bovine serum albumin (BSA) at a 5:1 molar ratio before treatment as previously described [19]. Solutions and reagents used for cell cultures were from GIBCO Life Technologies Ltd. (Grand Island, NY, USA). Antibodies used are from Cell Signaling Technology Inc. (Danvers, MA, USA). All other reagents and kits were purchased from Sigma Aldrich and Invitrogen (Carlsbad, CA, USA) unless otherwise noted.

### Cell culture and stimulation

HepG2 cells (ATCC, Manassas, VA, USA) were routinely cultured with Dulbecco’s Modified Eagle’s Medium containing 10% fetal bovine serum. 2 × 10^4^ cells were seeded into 24-well plates 24 h prior to treatments at approximately 60% confluence. After the starvation for 24 h, cells were exposed to different dose of PA for 24 h with or without the preincubation of 50 μM DHA for another 24 h. BSA and ethanol were used as controls. Cell viability after FFA treatments was monitored by trypan blue exclusion. No change in viability was observed with the concentrations used in this study. To knock down NLRC4 expression, HepG2 cells were transfected with 20 pmol NLRC4 siRNA, a commercially available small interfering RNAs (siRNAs) duplex components against this molecule (sc-60328A, sc-60328B and sc-60328 C; Santa Cruz, CA, USA) or a negative control siRNA (sc-37007; Santa Cruz, CA, USA), a RNA duplex with no known sequence homology, in a 24-well format using Lipofectamine 2000 (Invitrogen, Carlsbad, CA) according to the manufacturer's instructions.

### Observation of lipid accumulation

The lipid accumulation in HepG2 cells was evaluated by Oil Red O staining and the measurement of triglyceride (TG) content. Briefly, samples were fixed with 4% paraformaldehyde then stained with Oil Red O for 15 min. Then, the samples were counterstained with hematoxylin for 5 min. Results were examined by light microscopy. Intracellular TG content was evaluated after lysis of the cells with 1 × cell lysis buffer (1% Triton X-100, 150 mM NaCl, 10 mM Tris, pH 7.4, 1 mM EDTA, 1 mM EGTA, 0.2 mM phenylmethylsulfonylfluoride, 0.2 mM sodium orthovanadate, and 0.5% NP-40) (Promega, Madison, WI, USA). TG concentration was determined by the EnzyChrom^TM^ triglyceride assay kit (Bioassay Systems) according to the protocal provided by manufacturer and normalized by protein content.

### Measurement of inflammatory cytokines by ELISA assay

The cell culture media were centrifuged at 10,000 g for 10 min at 4°C and the supernatants were stored at −20°C before analysis. Secretory levels of inflammatory cytokines, including TNF-α, IL-1β, IL-18 and MCP-1 proteins, in cell-free culture supernatants were determined by Quantikine ELISA kit (R&D Systems Inc, Mineapolis, USA). The color generated was determined by measuring the OD value at 450 nm of spectrophotometric microtiter plate reader (Molecular Devices Corp., Sunnyvale, CA, USA). A standard curve was run on each assay plate using recombinant proteins in serial dilutions. The limits of sensitivity of the kits are 0.5, 7.0, 3.9 and 5.5 pg/mL for TNF-α, IL-1β, IL-18 and MCP-1, respectively.

### Real-time PCR assay of NLRC4 mRNA in HepG2 cells

Total RNA in cells was isolated using TRIzol reagent and reversely transcribed to cDNA using Super Script II Rnase H Reverse Transcriptase (Invitrogen, Carlsbad, Calif) according to the manufacturer’s instructions. The cDNA samples were stored at −20°C for real-time PCR. The PCR primers were designed by Premier Primer 5.0 software as the following: 5’-CCAGTCCCCTCACCATAGAAG-3’ (forward) 5’-ACCCAAGCTGTCAGTCAGACC-3’ (reverse) for NLRC4; 5’-CCTGGCACC- CAGCACAAT-3’ (forward) and 5’-GCCGATCCACACGGAGTACT-3’ (reverse) for β-actin. Real-time PCR was done with SYBR Green PCR Master Mix (Invitrogen, Carlsbad, Calif) using a 7500 Real-Time PCR System (Applied Biosystems). Melt curve analysis was included to assure a single PCR product was formed. Values were corrected using human β-actin gene. The relative NLRC4 mRNA levels were presented as percentage of that in control cells.

### Western blotting

Cellular protein was extracted with 1 × cell lysis buffer. Protein concentration was determined using the BCA^TM^ assay kit from Thermo Fisher Scientific Inc. (Huntsville, AL) according to the user manual. Protein (40 μg) from each sample was separated by 10-12% SDS-PAGE and electrotransferred to polyvinyl denedifluoride (PVDF) membranes. The membranes were blocked with 5% BSA in TBS for 1 h at room temperature and incubated overnight at 4°C using the following primary antibodies: 1:1000 rabbit anti β-actin, 1:500 NLRC4 (Santa Cruz, CA, USA), 1:1000 caspase-1 and 1:500 IL-1β, followed by 1:2000 dilution of goat anti-rabbit horseradish peroxidase-labeled antibody. The bands were visualized using the ECL system, and the band density was determined by Image J software (NIH, USA).

### Caspase-1 activity assay

HepG2 cells were homogenized in 1 × cell lysis buffer and caspase-1 activity was determined using colorimetric assay based on the cleavage of the substrate Aacetyl-Tyr-Val-Ala-Asp *p*-nitroanilide (Ac-YVAD-*p*NA) (R&D Systems, Minneapolis, MN, USA). 1 × 10^4^ cells were seeded into 96-well plates and treated in the same way as the method mentioned above. Then, cell-free culture media were analyzed with caspase-1 activity commercial kit according to the protocols provided by the manufacturer. Briefly, 90 μL standards, samples or control was added to the appropriate microtiter wells, and then 10 μL Ac-YVAD-*p*NA was pipetted into each well, and incubated for 2 h at 37°C. and the absorbance of each well at 405 nm was recorded after calibrating the plate reader against the chromogen blank. Cleavage of the substrate by caspase-1 releases *p*NA that can subsequently be quantified by the absorbance difference.

### Statistical analyses

All of the experiments were performed in triplicate and repeated at least three times. Data are expressed as mean ± SE. The significance of differences was determined by One-way ANOVA using SPSS13.0 software (SPSS, Chicago, IL). A value of *P* < 0.05 was considered to be statistically significant.

## Results

### DHA suppresses PA-induced lipids accumulation in HepG2 cells

**Figure 1:**
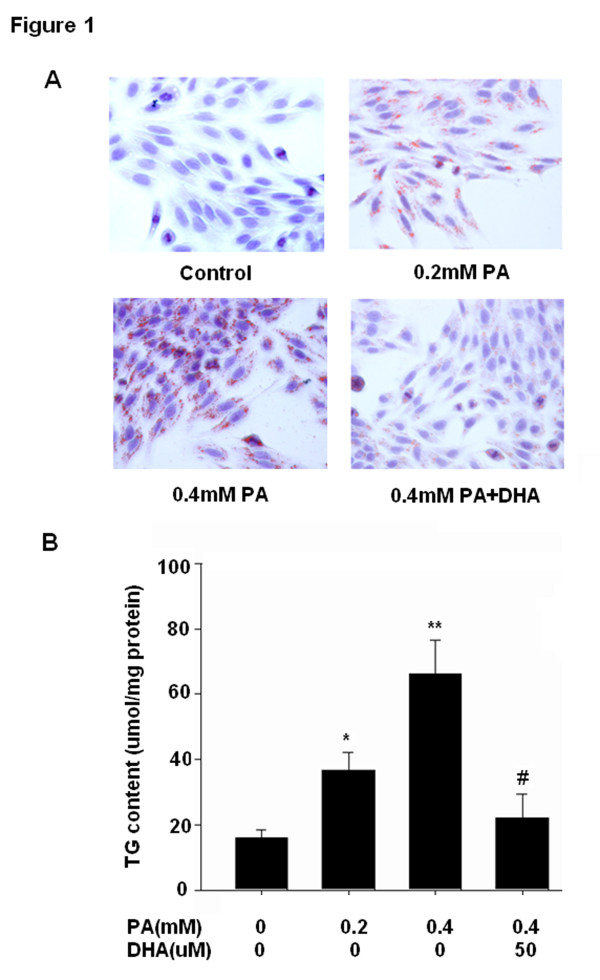
**DHA suppresses PA-induced lipids accumulation in HepG2 cells.** (**A**) Representative Oil Red O staining of cells with different treatments is shown. Cells were examined by light microscopy at a magnification of 400×; (**B**) Intracellular TG content was measured by an ELISA assay. TG concentration was normalized by protein content. Data are shown as the mean ± S.E.; n = 4. *P < 0.05, **P < 0.01 compared with untreated cells; #*P* < 0.01 versus 0.4 mM PA-treated cells.

We first analyzed the lipid accumulation qualitatively by Oil red O staining. As shown in Figure 1A, HepG2 cells treated for 24 h with PA exhibited significant lipid droplet accumulation dose-dependently compared with untreated cells. Preincubation with DHA significantly prevented 0.4 mM PA-induced lipid deposition and the most effective inhibition of lipid accumulation occurred at a dose of 50 μM. DHA alone or vehicle (ethanol) did not affect basal levels of lipid deposition (data not shown). To evaluate the effect of PA and DHA on lipids accumulation quantitatively, we next measured TG concentration in cells lysates. Consistently, treatment with PA resulted in a obvious increase in TG content compared to control cells, which was attenuated significantly by pretreatment with 50 μM DHA (Figure 1B). The lipid deposition and TG content in BSA carrier control cells were comparable with untreated cells (data not shown).

### DHA attenuates PA-induced inflammatory cytokines in HepG2 cells

**Figure 2:**
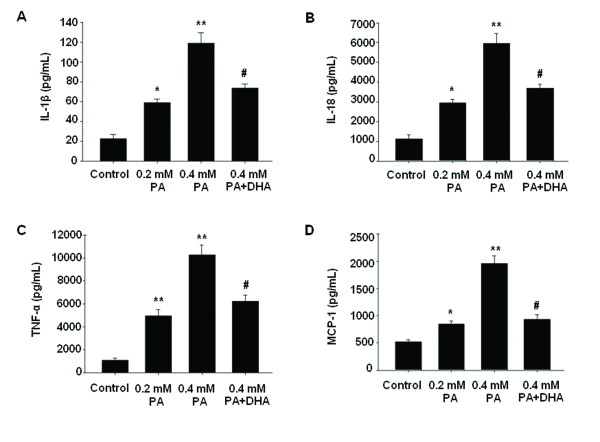
**DHA attenuates PA-induced inflammatory cytokines overproduction in HepG2 cells.** Cells were treated accordingly and then cell-free culture supernatants were assayed for IL-1β (**A**), IL-18 (**B**), TNF-α (**C**) and MCP-1 (**D**) by commercial ELISA kits. The data represent a mean of 4 experiments. *P < 0.05, **P < 0.01 compared with untreated cells; #*P* < 0.01 versus 0.4 mM PA-treated cells.

We examined the effects of PA on the productions of IL-1β, IL-18, TNF-α and MCP-1 in HepG2 cells. Figure 2 showed that PA exposure significantly increased the productions of all cytokines in a dose-dependent manner compared with the control cells. Pretreatment with 50 μM DHA for 24 h significantly alleviated PA-induced overproduction of all these inflammatory cytokines.

### DHA inhibits PA-induced NLRC4 mRNA expression

**Figure 3:**
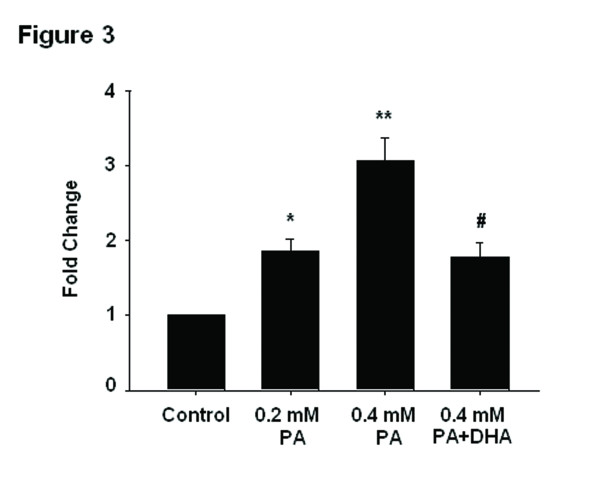
**DHA inhibits PA-induced NLRC4 mRNA expression.** Cells were treated accordingly and then NLRC4 mRNA was measured by real-time PCR The data are normalized using β-actin as control and expressed as fold over untreated cells. The experiment was repeated 4 times. *P < 0.05, **P < 0.01 compared with untreated cells; #*P* < 0.01 versus 0.4 mM PA-treated cells.

Since only the processed cytokines are biologically active, we hypothesized that the inflammasomes which are essential in regulating the proteolytic maturation of pro-IL-1β and other proinflammatory cytokines would involved in PA-induced inflammation. As shown in Figure 3, PA elicited the expression of NLRC4 mRNA in a dose-dependent manner. DHA pretreatment significantly decreased PA-induced NLRC4 mRNA expression.

### DHA reverses PA-induced lipid accumulation and inflammation through repressing NLRC4 expression

**Figure 4:**
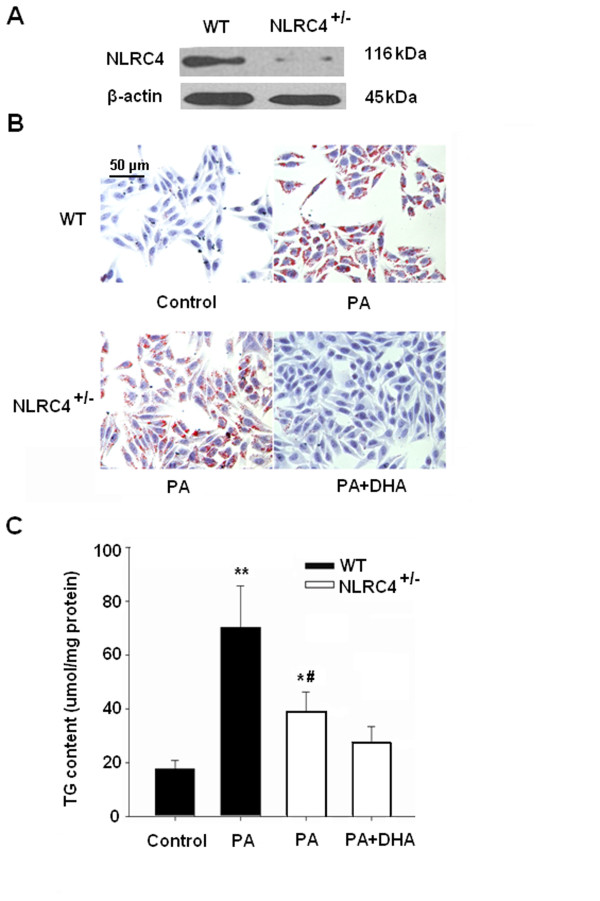
**DHA reverses PA-induced lipid accumulation through repressing NLRC4 expression.** (**A**) Generation of a HepG2 cell model with specific NLRC4 knockdown (NLRC4^+/−^). NLRC4 and β-actin protein levels were evaluated by immunoblotting. (**B**) Representative Oil Red O staining of wild-type (WT) and NLRC4^+/−^ HepG2 cells were treated with different dose of PA with or without DHA preincubation wild-type (WT) and NLRC4^+/−^ cells are shown. Cells were examined by light microscopy at a magnification of 400×; (**C**) Intracellular TG content was measured by an ELISA assay. TG concentration was normalized by protein content. Data are shown as the mean ± S.E.; n = 4. *P < 0.05, **P < 0.01 compared with untreated cells; #*P* < 0.01 versus 0.4 mM PA-treated cells.

**Figure 5:**
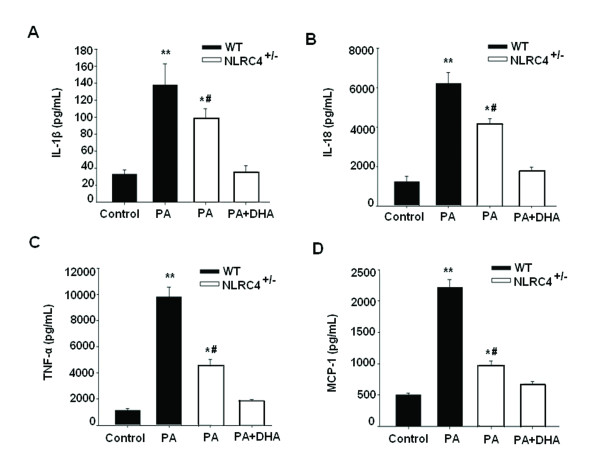
**DHA reverses PA-induced inflammation through repressing NLRC4 expression.** WT and NLRC4^+/−^ HepG2 cells were treated with different dose of PA with or without DHA preincubation and then cell-free culture supernatants were assayed for IL-1β (**A**), IL-18 (**B**), TNF-α (**C**) and MCP-1 (**D**) by commercial ELISA kits. The data represent a mean of 4 experiments. *P < 0.05, **P < 0.01 compared with untreated cells; #*P* < 0.01 versus 0.4 mM PA-treated cells.

To test whether NLRC4 might modulate PA-induced steatohepatitis, cells were transfected with NLRC4 siRNA (NLRC4^+/−^ cells). As shown in Figure 4A, the protein level of NLRC4 decreased dramatically by 91.2% by the transient RNA interference technology. Then, NLRC4^+/−^ cells were exposed to PA with or without DHA preincubation. Compared to the wild-type cells, the lipid deposition and TG content were significantly ameliorated in PA-treated NLRC4^+/−^ cells and were completely abolished by DHA preincubation (Figure 4B and C). Consistently, DHA almost abrogated PA-elicited production of IL-1β, IL-18, TNF-α and MCP-1 in NLRC4^+/−^ cells (Figure 5). No significant difference was observed in cells treated with the control siRNA compared with untreated cells (Additional file 1: Figure S1 and Table S1).

### DHA inhibits caspase-1 activity and IL-1β maturation

**Figure 6:**
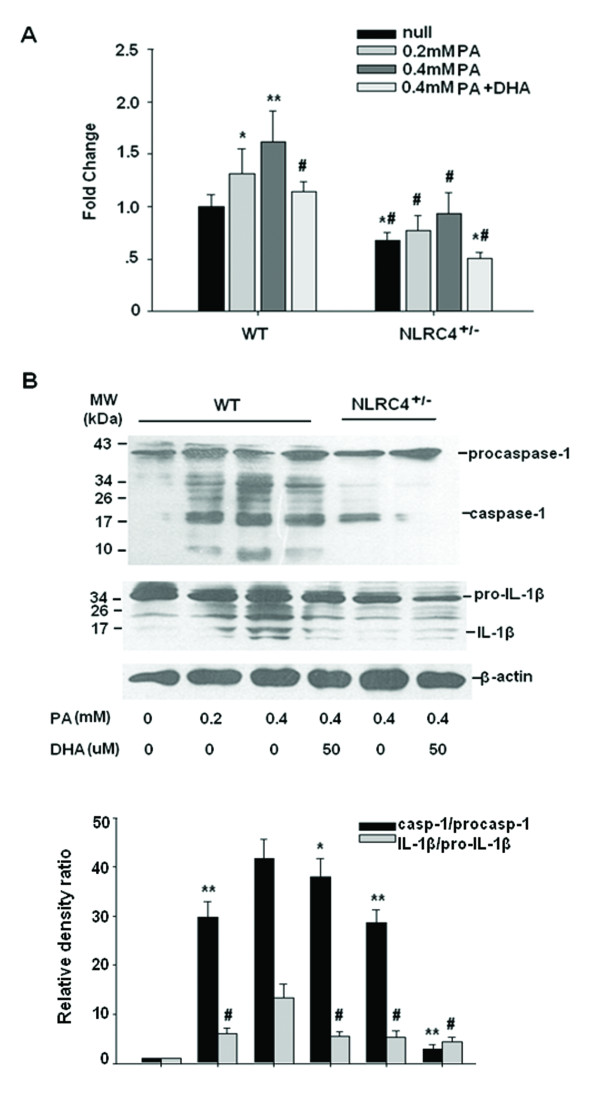
**DHA inhibits caspase-1 activity and IL-1β maturation.** WT and NLRC4^+/−^ HepG2 cells were treated with different dose of PA with or without DHA preincubation and cells were homogenized in lysis buffer. (**A**) Caspase-1 activity was determined using colorimetric assay based on the cleavage of substrate Ac-YVAD-pNA. The results were expressed as fold over untreated WT cells. *P < 0.05, **P < 0.01 compared with untreated cells; #*P* < 0.01 versus 0.4 mM PA-treated cells. (**B**) Immunoblot analysis showing caspase-1 cleavage and active IL-1β accumulation. This gel is the representative image of three independent experiments (upper panel). Quantified data of protein levels indicates in lower panel. The values of density of proteins were all justified with β-actin. The relative density ratios of untreated WT cells were set at a value of 1.0. The values represent the mean ± S.E (*P < 0.05, **P < 0.01 compared with WT cells treated with 0.4 mm PA for the ratio of caspase-1/procaspase-1; #*P* < 0.01 versus 0.4 mM PA-treated WT cells for the ratio of IL-1β/pro-IL-1β).

Conversion of the inactive pro-IL-1β to its mature form IL-1β requires the proteolytic action of IL-1β-converting enzyme (ICE), which is also termed as caspase-1. The enzyme is activated by inflammsomes, such as NLRP3 and NLRC4 [17]. We tested the effect of DHA on caspase-1 activity by an ELISA assay. In the presence of different dose of PA, caspase-1 activity increased accordingly whereas partially reversed by DHA preincubation. Compared to PA-treated wild-type cells, the knockdown of NLRC4 led to a more depressed caspase-1 activity in each experimental treatment. Furthermore, DHA preincubation completely reversed PA-induced caspase-1 activation in NLRC4^+/−^ HepG2 cells (Figure 6A).

To determine the effect of DHA on IL-1β maturation, we measured the procaspase-1 and pro-IL-1β processing by western blotting assay. As presented in Figure 6B, PA elicited caspase-1 and IL-1β release dose-dependently compared with control cells. DHA reduced PA-induced procaspase-1 and pro-IL-1β processing significantly. NLRC4 siRNA repressed both caspase-1 and IL-1β release induced by 0.4 mM PA, and these events were potentiated by the preincubation of DHA.

## Discussion

NAFLD encompasses four histopathology stages: simple steatosis, steatosis with inflammation termed as NASH, cirrhosis and hepatocellular carcinoma [20]. The steatosis and inflammation in NASH often result in the following more severe liver injuries [21]. Hence, elucidation of the possible mechanisms responsible for NASH and impeding the progress of NASH are of emerging importance. DHA possesses hepatoprotective effects and DHA supplementation decreases liver fat content in children with NAFLD [16,22]. In the present study, we reported that DHA ameliorates PA-induced lipid accumulation, inflammatory cytokines production in HepG2 cells. Further experiments indicated that DHA down regulates NLRC4 mRNA expression and caspase-1 activity, resulting in the reduction of IL-1β release. These data suggested that DHA exerts protective effects on NASH not only at the 1^st^ hit but also at the 2^ed^ one.

The development of NASH has been initially linked to increased lipids deposition and plasma concentrations of FFAs are elevated in patients with NASH [23,24]. Previous study has suggested that FFAs induce steatohepatitis by participating in lipotoxic responses and chronic inflammatory responses [25,26]. It has also been suggested that saturated FFAs such as laurate and PA increases lipid accumulation and induces a pro-inflammatory response [19,27]. In this study, we chose a molar ratio 5:1 of PA to BSA to mimic the pathophysiological states in which unbound FFA concentrations are high [28]. Our data demonstrates that exposure of HepG2 cells to PA results in lipid accumulation and the overproduction of inflammatory cytokines in a dose-dependent manner. This is in agreement with a previous study that the serum concentrations of FFAs were increased in patients with NAFLD and correlated with the development of more severe liver disease [29]. Importantly, recent evidence showed that saturated fatty (palmitic) acid activated the inflammasome and triggered release of danger signals from hepatocytes in a caspase-1-dependent manner [30], suggesting that the activation of inflammasomes also may contribute to the development of NASH. In the present study, we demonstrated that PA activated NLRC4 inflammasome function, indicated by cleavage of procaspase-1 and increased IL-1 production, along with the increased expression of NLRC4 mRNA. This novel observation differs from a previous report that NLRC4 had no effect on PA-induced IL-1β or IL-18 production in macrophages [17]. The discrepancy may represent cell-specific differences in response to PA between hepatic cells (HepG2) and macrophages or pathways by which pro-IL-1β was cleavaged [31]. Interestingly, the knockdown of NLRC4 has not completely inhibited PA-induced IL-1β production. This observation complements a previous report that NALP3, another inflammasome, is involved in PA-induced IL-1β overproduction in hepatocytes [30].

Our study clearly demonstrates that preincubation with DHA significantly attenuates the lipid accumulation and inflammatory cytokines overproduction in cells exposed to excess PA. It was consistent with a previous study that DHA alleviates lipid deposition and inflammation and the supplementation of n-3 PUFAs ameliorates NAFLD in Japanese men and women [32]. However, the exact mechanisms of DHA in preventing NASH are not yet clear. Recent studies demonstrated that DHA inhibits lipid accumulation by coordinately suppressing lipid synthesis in the liver by impairing the proteolytic release of SREBP-1c and/or by suppressing SREBP-1c gene expression and upregulating fatty acid oxidation by serving as an in vivo activator of peroxisome proliferator-activated receptor alpha (PPAR-α) [33]. DHA was also found to exert anti-inflammatory effects by decreasing the production of inflammatory eicosanoids [34], reactive oxygen species production [35] and the expression of adhesion molecules [36,37] or giving rise to a family of anti-inflammatory mediators termed resolvins [38]. Our data suggested that DHA preincubation inhibited PA-induced NLRC4 inflammasome activation and decreased the cleavage of procaspase-1 and IL-1 production. Further, knockdown of NLRC4 significantly inhibited PA-induced lipid accumulation and almost completely abolished that in the presence of DHA. To our best knowledge, we show here for the first time that DHA ameliorates NASH through suppressing the NLRC4 inflammasome activation.

Our current study shows that PA triggered caspase-1 activity in a dose-dependent manner whereas DHA inhibited PA-induced caspase-1 activation, leading to the decreased cleavage of pro-IL-1β and IL-1β production. IL-1β has been implicated in the pathogenesis of steatohepatitis [39]. Unlike other secreted proteins, IL-1β is synthesized as a precursor protein pro-IL-1β, whose multiple extracellular activities in response to various proinflammatory stimuli are attributed to receptor binding of IL-1β itself [31,40]. It is generally accepted that cleavage of pro-IL-1β is mediated by inflammasomes [41] and conversion of the inactive pro-IL-1β to IL-1β requires the proteolytic action of caspase-1 [42]. It was suggested that the specific inhibition of caspase-1 by the caspase inhibitor blocked the maturation of IL-1β [43] and no significant IL-1β produced in PA-treated macrophages derived from caspase-1^−/−^ mice [17]. We showed an inhibitory effect of DHA on caspase-1 activity and IL-1β maturation in HepG2 cells, an effect which, to our best knowledge, has not been reported previously.

Inflammation is a response to lipid accumulation and can accelerate the injury. Treatment of mice with IL-1β decreased fatty acid metabolism, resulting in hepatic triglyceride storage [44]. In the present study, we found that knockdown of NLRC4 inhibited PA-induced inflammation, thereby further attenuating lipid accumulation in cells. This is agreement with previous studies that IL-1β stimulation exacerbates lipid accumulation in hepatic cells and fatty livers of apolipoprotein E knockout mice [45] and enhanced liver injury induced by inflammatory stress was observed in NASH [46]. Thus, exposure of DHA is effective for preventing and reversing lipid accumulation and the inflammatory response which may accelerate the lipid metabolism disorder and/or more severe liver injuries.

In summary, our present findings clearly show that PA triggers lipid accumulation and inflammatory cytokines overgeneration in HepG2 cells, which is ameliorated by DHA through attenuating NLRC4 inflammasome activation, caspase-1 activation and IL-1β cleavage. Taken together, these data suggests that DHA may server as a potential nutrient antagonist to NLRC4 and our findings offer new insights into the potential mechanisms of action of DHA in mediating the beneficial hepatoprotective effects in NASH.

## Abbreviations

DHA: Docosahexaenoic acid; PUFAs: Polyunsaturated fatty acids; NASH: Nonalcoholic steatohepatitis; IL-1β: Interleukin-1β; TG: Triglyceride; PA: Palmitate; NAFLD: Non-alcoholic fatty liver disease; FFAs: Free fatty acids; Ac-YVAD-pNA: Aacetyl-Tyr-Val-Ala-Asp p-nitroanilide.

## Competing interests

The authors declare that they have no competing interests.

## Authors’ contributions

XQL and WHL contributed to design the study, analysis and interpretation of data, and drafting of the manuscript. YY, MX, YJX and TBZ assisted with interpretation of the result and critical revision of the manuscript. TRS and XLT carried out the ELISA studies. All authors have given their final approval of the submitted version of the manuscript.

## Supplementary Material

Additional file 1 **Figure S1.Representative Oil Red O staining of cells with different treatments is shown.** Cells were transiently transfected with 20 pmoL control siRNA or NLRC4 siRNA for 48 h and examined by light microscopy at a magnification of 400×. The images are representative of typical staining. **Table S1.Effects of siRNA transfection on the production of cytokines (mean ± SE; pg/mL) in HepG2 cells.** Cells were transiently transfected with 20 pmoL control siRNA or NLRC4 siRNA for 48 h. Then the cell-free culture supernatants were centrifuged at 10,000 g for 10 min at 4°C and the supernatants were stored at -20°C before assaying IL-1β, IL-18, TNF-α and MCP-1 by commercial ELISA kits. The data represent a mean of 4 experiments. Click here for file
