# Supplementary material for: Docosahexaenoic acid ameliorates palmitate-induced lipid accumulation and inflammation through repressing NLRC4 inflammasome activation in HepG2 cells
Source: Nutr Metab (Lond). 2012 Apr 19;9:34. doi: 10.1186/1743-7075-9-34 (PMC3428681; doi:10.1186/1743-7075-9-34)
Supplement: Additional file 1 — Figure S1.Representative Oil Red O staining of cells with different treatments is shown. Cells were transiently transfected with 20 pmoL control siRNA or NLRC4 siRNA for 48 h and examined by light microscopy at a magnification of 400×. The images are representative of typical staining. Table S1.Effects of siRNA transfection on the production of cytokines (mean ± SE; pg/mL) in HepG2 cells. Cells were transiently transfected with 20 pmoL control siRNA or NLRC4 siRNA for 48 h. Then the cell-free culture supernatants were centrifuged at 10,000 g for 10 min at 4°C and the supernatants were stored at -20°C before assaying IL-1β, IL-18, TNF-α and MCP-1 by commercial ELISA kits. The data represent a mean of 4 experiments. [file 1743-7075-9-34-S1.doc]

**Supplemental Figure 1**

**
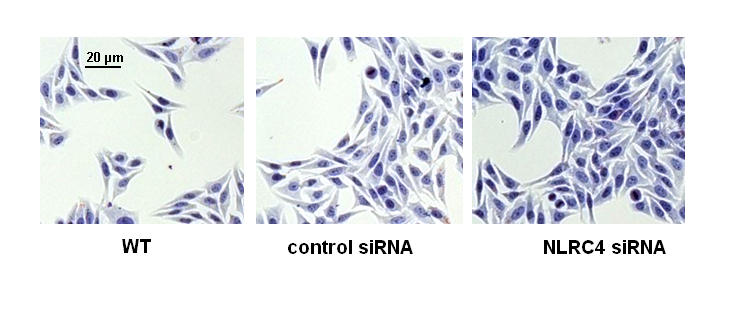
**

**Figure legend:** Representative Oil Red O staining of cells with different treatments are shown. Cells were transiently transfected with 20 pmoL control siRNA or NLRC4 siRNA for 48 h and examined by light microscopy at a magnification of 400×. The images are representative of typical staining.

**Supplemental Table I Effects of siRNA transfection on the production of cytokines (mean ± SE ;pg/mL) in HepG2 cells.** Cells were transiently transfected with 20 pmoL control siRNA or NLRC4 siRNA for 48 h. Then the cell-free culture supernatants were centrifuged at 10,000 g for 10 min at 4°C and the supernatants were stored at -20°C before assaying IL-1β, IL-18, TNF-α and MCP-1 by commercial ELISA kits. The data represent a mean of 4 experiments.

| Group | IL-1β | IL-18 | TNF-α | MCP-1 |
| --- | --- | --- | --- | --- |
| WT | 33.2 ± 5.7 | 1214.4 ± 289.2 | 1150.3 ± 180.8 | 501.6 ± 33.8 |
| Control siRNA | 35.5 ± 6.4 | 1201.4 ± 254.9 | 1178.3 ± 189.2 | 519.5 ± 54.6 |
| NLRC4 siRNA | 33.8 ± 5.7 | 1192.5 ± 237.2 | 1140.8 ± 157.1 | 488.3 ± 42.5 |
